# Human Liver Sinusoid on a Chip for Hepatitis B Virus Replication Study

**DOI:** 10.3390/mi8010027

**Published:** 2017-01-20

**Authors:** Young Bok (Abraham) Kang, Siddhartha Rawat, Nicholas Duchemin, Michael Bouchard, Moses Noh

**Affiliations:** 1 Mechanical Engineering and Mechanics, Drexel University, Philadelphia, PA 19104, USA; heavenboki@gmail.com; 2 Graduate Program in Molecular and Cellular Biology and Genetics, Graduate School of Biomedical Sciences and Professional Studies, Drexel University College of Medicine, Philadelphia, PA 19129, USA; rawat.sid@gmail.com (S.R.); nd397@drexel.edu (N.D.); 3 Department of Biochemistry and Molecular Biology, Drexel University College of Medicine, Philadelphia, PA 19129, USA

**Keywords:** liver sinusoid, human-liver-sinusoid-on-a-chip, Hepatitis B virus (HBV), HBV replication study, hepatocyte, microfluidic platform

## Abstract

We have developed a miniature human liver (*liver-sinusoid-on-a-chip*) model using a dual microchannel separated by a porous membrane. Primary human hepatocytes and immortalized bovine aortic endothelial cells were co-cultured on opposite sides of a microporous membrane in a dual microchannel with continuous perfusion. Primary human hepatocytes in this system retained their polygonal morphology for up to 26 days, while hepatocytes cultured in the absence of bovine aortic endothelial cells lost their morphology within a week. In order to demonstrate the utility of our human-liver-sinusoid-on-a-chip, human hepatocytes in this system were directly infected by Hepatitis B Virus (HBV). Expression of the HBV core antigen was detected in human hepatocytes in the microchannel system. HBV replication, measured by the presence of cell-secreted HBV DNA, was also detected. Importantly, HBV is hepatotropic, and expression of HBV RNA transcripts is dependent upon expression of hepatocyte-specific factors. Moreover, HBV infection requires expression of the human-hepatocyte-specific HBV cell surface receptor. Therefore, the ability to detect HBV replication and Hepatitis B core Antigen (HBcAg) expression in our microfluidic platform confirmed that hepatocyte differentiation and functions were retained throughout the time course of our studies. We believe that our human-liver-sinusoid-on-a-chip could have many applications in liver-related research and drug development.

## 1. Introduction

The World Health Organization has estimated that approximately 240 million people in the world have a chronic Hepatitis B Virus (HBV) infection of the liver [1,2]. A chronic HBV infection has been linked to the development of severe liver diseases, including hepatitis, cirrhosis, and hepatocellular carcinomas [3]. Liver inflammation associated with immune-mediated destruction of HBV-infected hepatocytes, integration of the HBV genome into the host genome, and the activities of HBV genome-encoded proteins have all been proposed as potential mechanisms that underlie the development of HBV-associated diseases; however, there are still significant gaps in our understanding of how a chronic HBV infection can lead to the development of severe liver diseases [4,5]. One reason for this gap in our knowledge is the absence of in vitro human hepatocyte culture systems that mimic the environment and cellular architecture of the liver and can be used for long-term studies of the consequence of an HBV infection to human hepatocyte physiology. The development of these types of systems is critical for providing platforms that can be used to define HBV effects on hepatocyte physiology.

Many studies intent on understanding the biology of liver diseases and liver damage by toxins or infectious agents have been carried out using animal models or established cell lines due to the limited availability of normal human liver cells [6]. Moreover, because the host range of HBV is very narrow, the available animal models that can be directly infected by HBV are limited; for example, mice and rats cannot be directly infected by HBV [7,8]. Unfortunately, expression of the recently discovered human HBV receptor in rodent hepatocytes does not render these susceptible to an HBV infection [9]. Although in vitro liver cell culture models exist, these systems typically consist of immortalized hepatocyte cell lines that do not represent authentic differentiated hepatocytes and typically have altered signaling pathways [10,11]. As a result, attempts to analyze the consequences of an HBV infection may not be accurately represented in these in vitro systems. Unfortunately, primary hepatocytes quickly de-differentiate when hepatocytes are isolated from the liver and cultured in traditional cell-culture formats, limiting their use in long-term studies [12,13]. Moreover, these conventional cell culture platforms, containing only hepatocytes, do not provide a physiological environment where hepatocytes constantly interact with other types of liver cells. Hence, there remains a need to develop an in vitro primary human liver model that is a physiologically relevant model for liver research.

**Figure 1:**
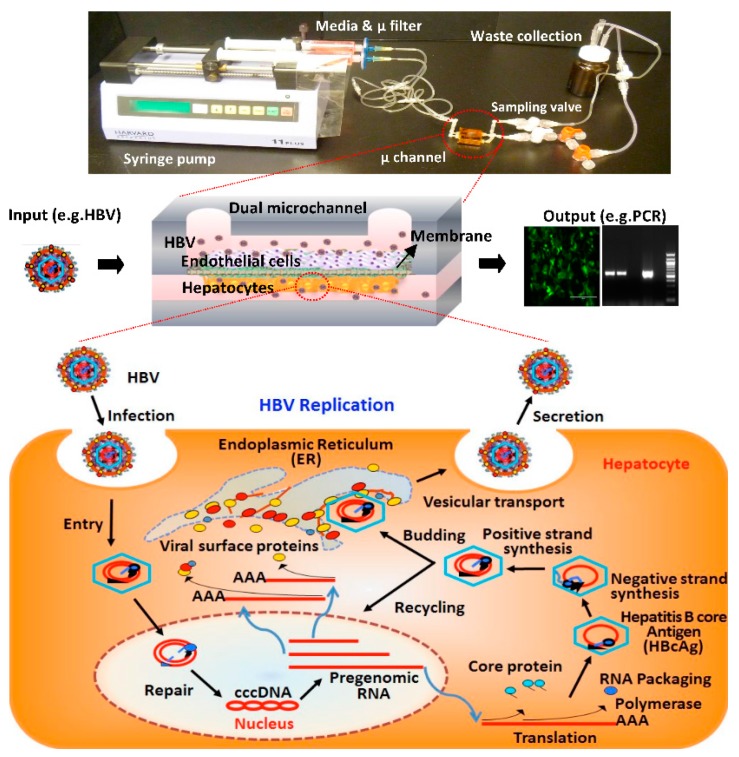
In vitro liver model using a microfluidic cell culture platform with a continuous perfusion system and Hepatitis B Virus (HBV) replication cycle. Hepatocytes cultured in the liver sinusoid on a chip with a perfusion system are infected with HBV. HBV replicates in the hepatocytes and is secreted from the host cell. The expression of HBV proteins and replication of HBV in infected cells can be detected through immunofluorescence staining or polymerase chain reaction (PCR) analysis, respectively.

Many attempts have been made with the help of microfabrication and microfluidics technologies to develop more accurate in vitro human liver models that can recapitulate the in vivo microenvironment and architecture of the liver [14,15,16,17,18]. However, none of these have yet been able to successfully facilitate long-term liver-related disease studies. In our previous studies, we successfully demonstrated a microchannel-based in vitro assay for studying HBV replication in a single-layer microchannel containing primary rat hepatocytes or HepG2 cells under static culture conditions [19]. More recently, we presented a rat liver sinusoid model that consisted of two microchannels simulating the blood sinusoid and bile duct (Figure 1) [20]. The two microchannels were separated by a porous membrane with hepatocytes and endothelial cells cultured on the opposite sides of the membrane. We demonstrated the long-term (over 30 days) maintenance of primary rat hepatocytes differentiation and functions when the hepatocytes were co-cultured with endothelial cells. We also used this system as a novel platform for studying HBV replication, although the initial infection of the rat hepatocytes by HBV could not be studied because rat hepatocytes are not directly susceptible to HBV infection and this initial stage of infection had to be bypassed by delivering the HBV genome to the hepatocytes via a recombinant adenovirus containing a replication-competent copy of the HBV genome.

Our goal in the present study was to show that human hepatocytes could be co-cultured with endothelial cells in a similar microfluidic platform as in our previous studies, and that human hepatocytes in this system retain their differentiation status and functions for long-term culture and can be directly infected by HBV. To this end, we present a human-liver-sinusoid-on-a-chip model that is created by extending our dual microchannel platform with primary rat hepatocytes to a primary human hepatocyte culture system. We further demonstrate the utility of our liver model by demonstrating that human hepatocytes in this system can be directly infected with HBV (Figure 1).

Human hepatocytes are the natural site of an HBV infection, and, although there are many aspects of the HBV lifecycle that are understood, there are still major gaps in our understanding of precisely how HBV infects hepatocytes and replicates in these cells (Figure 1). An impediment to fully understanding all aspects of the HBV lifecycle has been the paucity of available human hepatocyte systems that allow for a direct HBV infection that can be monitored and analyzed for a longer time than in conventional primary hepatocyte culture systems. The recent identification of the human-hepatocyte cell-surface HBV receptor is likely to further enhance our understanding of mechanisms that regulate HBV entry into hepatocytes [21]. The HBV genome is a highly compact, partially double stranded, relaxed circular DNA that is delivered to the nucleus where it is converted into covalently closed circular DNA (cccDNA) by the host-cell DNA repair machinery [3,7,22,23]. The HBV cccDNA is used as the template for transcription of HBV mRNAs, which are transported to the cytoplasm and translated to generate HBV proteins [1,23,24]. One of the HBV mRNAs, the pregenomic mRNA (pgRNA), is encapsidated into viral capsids that are made of the HBV core protein, where the pgRNA is reverse transcribed to generate the viral DNA genome [24,25]. Viral surface proteins (HBsAg) synthesized on the endoplasmic reticulum envelop the viral capsids, and the virus is then secreted from the host cell [24,25]. While anti-HBV therapies are available, none are curative. Currently available anti-HBV drugs cannot completely cure an HBV infection because none target cccDNA, and the stability of the cccDNA is such that once anti-HBV therapies are terminated, there is often a quick rebound in viral replication [26,27,28]. Due to the rapid de-differentiation of primary hepatocytes, the currently available cultured human hepatocyte model systems cannot be used to study methods for eliminating HBV cccDNA in hepatocytes. There is the need for novel human liver model systems in which the long-term culture of differentiated human hepatocytes can be used to assess methods or drugs that could eliminate cccDNA in HBV-infected hepatocytes.

In our studies using our human-liver-sinusoid-on-a-chip model, we have demonstrated the long-term maintenance of differentiated human hepatocytes as well as the utility of our system for direct HBV infection. We believe that our human liver model mimics the in vivo liver sinusoid and can be used for diverse liver biology studies and liver-related disease research such as the effects of an HBV infection on normal human hepatocyte physiology. Most importantly, this system could facilitate longer term studies in human hepatocytes that were previously not possible due to the rapid de-differentiation of primary human hepatocytes cultured in traditional cell-culture platforms.

## 2. Materials and Methods

### 2.1. Fabrication of Microfluidic Platforms

Templates for rectangular microchannels (approximately 15 mm long, 1 mm wide, and 600–800 μm high) were fabricated via the stereolithography technique with PhotoSilver 130 (EnvisionTEC GmbH, Gladbeck, Germany) resin and the Envision Tec^®^ Perfectory SXGA+ Standard UV Stereolithography system [20,29]. Polydimethylsiloxane (PDMS) microchannels were then made by the replica-molding method using the 3-dimensional (3D)-printed microchannel templates [19]. Two PDMS microchannels were treated with air plasma at 65 W for 70 s (Femtoscience, Yongin, Korea) and bonded together with a microporous polyethyleneterephthalate (PET) membrane (10 μm thick with a pore size of 0.4 μm) placed between them (Figure 1) [29,30]. The bonded dual-channel was wrapped with Kapton tape (Micronova, Torrance, CA, USA) for complete sealing. Inlet and outlet ports of the microchannels were made by implanting silicone tubes during PDMS replica molding. The bottom surface of the microchannel and the PET membrane were coated with collagen type I, and the assembled microchannel device was then sterilized using ultraviolet (UV) light as previously described [20].

### 2.2. Layered Co-Culture of Primary Human Hepatocytes and Endothelial Cells

Normal primary human hepatocytes in suspension were obtained through the Liver Tissue Cell Distribution System, Pittsburgh, Pennsylvania, which is funded by National Institutes of Health Contract #HHSN276201200017C. The cells were cultured in Dulbecco’s Modified Eagle Media (DMEM, Cellgro, Mediatech, Manassas, VA, USA), supplemented with 1 mM sodium pyruvate (Cellgro), 4 µg/mL insulin-transferrin-selenium (ITS, Gibco, Thermo Fisher Scientific, Waltham, MA, USA), 5 µg/mL hydrocortisone (HC, Sigma, St. Louis, MO, USA), 5 ng/mL epidermal growth factor (EGF, BD Sciences, San Jose, CA, USA), 1% penicillin-streptomycin, and 10% (*v*/*v*) fetal bovine serum (FBS, Gemini Bioproducts, West Sacramento, CA, USA) at 37 °C in 5% CO_2_.

Bovine aortic endothelial cells (BAECs) are immortalized, microvascular, endothelial cells that can be passaged repeatedly [31]. Because these cells are more readily available than primary human liver sinusoidal endothelial cells, we chose to use BAECs in our system as a proof-of-concept, wherein co-culture primary human hepatocytes and endothelial cells facilitated long-term survival and maintenance of hepatocyte differentiation. The BAECs were a kind gift from Dr. Robert Levy (Children’s Hospital of Philadelphia) [30]. The isolation and characterization of BAECs have been previously described [31]. BAECs were cultured in DMEM, supplemented with 10% (*v*/*v*) FBS and 1% (*v*/*v*) penicillin-streptomycin (Cellgro) at 37 °C in 5% CO_2_. For the layered co-culture, approximately 1 × 10^4^–2 × 10^4^ primary human hepatocytes with 70%–100% viability were plated in the collagen-coated microchannel using a pipette by slowly adding them from one side of the microchannel until the fluid began to exit from the other side of the microchannel. Cells adhered to the microporous membrane and formed a confluent monolayer by 24 h after plating, referred to as Day 1. The device was then flipped over and endothelial cells were plated on the opposite side of the microporous membrane and allowed to adhere for another 24 h. The device was then connected to a syringe pump to provide continuous perfusion of the culture medium with a flow rate of 30–40 µL/h (Figure 1). The flow rate was determined based on both the experimental observation of cell morphology under a range of flow rate 0–100 µL/h and the estimated shear stress as described in our previous study [20]. Multiple valves were placed downstream of the microchannel outlet to facilitate sampling of the flow-through medium. When not sampled, the culture medium coming out of the microchannel was collected into a waste bottle.

### 2.3. Live-Dead Staining and Imaging

The viability of primary human hepatocytes cultured in the microchannel was examined using a LIVE/DEAD Viability/Cytotoxicity Kit according to the manufacturer’s directions (ThermoFisher, Waltham, MA, USA). Briefly, the primary human hepatocytes were stained with calcein-AM (5 mg/mL) and ethidium bromide (2.5 mg/mL) fluorescent stains for 15 min at 37 °C. Images were acquired using an inverted fluorescence microscope.

### 2.4. Source, Purification, and Quantification of Infectious HBV

Infectious HBV was isolated from the cell-culture supernatants of the HBV-producing cell lines, HepG2.215 or HepAD38 cells [32,33]. HBV-producing cell lines were seeded at 80% confluency and maintained for 7–10 days with a replenishment of medium every 2–3 days. Virus containing cell-culture supernatants were pooled and then cleared of cellular debris by centrifugation at 3000× *g* for 10 min. HBV was precipitated from pooled supernatants as described previously [34], with some modifications. Briefly, supernatants were polyethylene glycol 8000 (PEG8000) precipitated overnight at 4 °C, followed by centrifugation at 3000× *g* for 30 min to pellet PEG8000-precipitated material. Pellets containing infectious HBV were resuspended in DMEM at a volume equivalent to 1/10 of the starting cell-culture supernatant volume. A small portion of the concentrated stock was set aside and subjected to a standard polymerase chain reaction (PCR) with HBV-specific primers, following isolation of HBV DNA using the QIamp DNA Blood Mini Kit (Qiagen, Hilden, Germany) according to the manufacturer’s instructions. PCR analysis of dilutions of the plasmid pGEMHBV, which contains a DNA copy of the HBV genome, was used to create a standard curve for calculating genome equivalents (GE) present in precipitated HBV stocks; GE were calculated by comparing the intensity of viral stock PCR products observed following electrophoresis in an agarose gel to the intensity of PCR products in the standard curve.

### 2.5. Infection of Primary Human Hepatocytes with HBV or Recombinant Adenoviruses

Hepatocytes are naturally infected with HBV that is introduced into the liver sinusoid via the blood stream (Figure 1); however, because our goal was to demonstrate that hepatocytes cultured in our system can be directly infected by HBV, we directly introduced HBV into the bottom channel, where hepatocytes are cultured in order to improve the efficiency of viral infection. In a natural infection, HBV likely passes through the fenestrae of liver sinusoid endothelial cells (LSECs) to directly infect hepatocytes, and there is no evidence that HBV is transported through LSECs to infect hepatocytes. Hence, the direct infection method that we have used is consistent with a natural HBV infection. Infection of primary human hepatocytes in microchannels was carried out using concentrated HBV stocks, as described previously [35], at a genome equivalent of 250–500 per cell. An infection medium containing HBV, 2% DMSO and 4% PEG8000 was added to the microchannel without the introduction of flow for 16–18 h, and then the cells were washed three times with phosphate-buffered saline (PBS) and maintained in growth medium containing 2% DMSO [35]. Recombinant adenoviruses encoding either a humanized recombinant green fluorescent protein (hrGFP) alone adenovirus encoding green fluorescent protein (AdGFP) or hrGFP and a cDNA of the HBV genome (AdGFP-HBV) were used to demonstrate that the hepatocytes were susceptible to a viral infection and capable of expressing HBV proteins. Due to the extensive overlap of open reading frames and the condensed nature of the HBV genome, the cDNA of HBV contains all HBV genes expressed from transcription promoters that are also part of the cDNA and hence require maintenance of the hepatocyte differentiation status for expression. The construction and use of these adenoviruses has been previously described [36]. For infection, 30–40 μL of the adenovirus solution was added slowly from one end of the microchannel until the solution began to exit the other end of the microchannel. The primary human hepatocytes were incubated with the recombinant adenovirus without introduction of flow for 16–18 h and washed as described above for the direct HBV infection. Fresh primary human hepatocyte medium was then introduced to the microchannels. The liver-sinusoids-on-a-chip with either AdGFP- or AdGFP-HBV-infected hepatocytes was connected to a perfusion system and maintained at 37 °C in 5% CO_2_. Expression of HBV core antigen was assessed approximately 10–14 days after AdGFP-HBV infection.

### 2.6. Analysis of Secreted HBV DNA

For HBV studies, we used the microchannel devices containing the layered co-culture of primary human hepatocytes and BAECs; hepatocytes were infected with HBV as described above. The medium from the HBV-infected and control uninfected hepatocytes in microchannels was collected at the indicated time points and stored at −80 °C for detection of HBV genome by PCR, as described below. Fluid samples of 1–2 mL were collected from each microchannel over the course of an entire day; PEG8000/1.5 M NaCl (30% (*w*/*v*)) was then added for the precipitation of secreted HBV. The final concentration of PEG8000 in a mixed sample was 8% (*w*/*v*). After incubation of these samples at 4 °C for approximately 16 h, HBV virions were obtained by centrifugation of the samples at 15,000× *g* for 15 min, followed by resuspension of the pellet in 50 μL PBS. The pelleted virions were then incubated with 1.5 μL of DNase I (10 mg/mL) and 0.5 μL of 1 M MgCl_2_ for one hour at 37 °C and then mixed with 5 μL of 0.5 M EDTA, 10 μL of 10% SDS, 0.2 μL of 2 M CaCl_2_, and 2.5 μL of proteinase K at 10 mg/mL. After incubation for 1–2 h at 55 °C, the samples were centrifuged at 15,000× *g* for 15 min and supernatants were mixed with 0.3 μL of 3 M sodium acetate (pH 4.8–5.2) and 75 μL of chilled 100% ethanol and stored at −20 °C overnight. The HBV genome was precipitated by a 15 min centrifugation at 15,000× *g*, and the pellet was resuspended in 20 μL of deionized water. The pelleted HBV genome was then analyzed by PCR using HBV-specific primers (568 bp product) as previously described [19,20]. As a positive control for detection of the HBV genome, a plasmid including the HBV genome was used.

### 2.7. Immunofluorescence Assay

After culturing HBV or recombinant adenovirus infected primary human hepatocytes for the indicated times, the dual microchannel was disconnected from the perfusion system and disassembled for assessment of the HBV core protein expression by immunofluorescence. The PET membrane that cells adhered to was placed on a microscope glass slide. The primary human hepatocytes cultured on the PET membrane were washed and incubated with 95% ethanol and 5% acetic acid mixture at −20 °C overnight to permeabilize and fix the cells. The cells were then washed twice with 2% bovine serum albumin (BSA) in PBS solution and then dried for 20 min after the 2% BSA solution was removed. The cells were incubated for 1 h at room temperature with an anti-HBV core antibody (HBcAb, SC-23945, Santa Cruz Biotechnology, Dallas, TX, USA) diluted in 2% BSA. After incubation, the cells were washed four times with 2% BSA in PBS. The cells were then incubated with a donkey anti-mouse IgG (Alexa fluor 594, Cat #A21203, Life Technologies, Carlsbad, CA, USA) for 1 h at room temperature, followed by three washes with PBS and one with water. Mounting medium including 4′,6-diamidino-2-phenylindole (DAPI) nuclear staining dye was added to the cells, and the cells were observed with a fluorescence microscope.

### 2.8. Image Assay

The average fluorescence intensity of the virus-infected cells from fluorescent microscopy images was quantified using ImageJ (NIH, Bethesda, MD, USA). Briefly, the background of images was subtracted, the image contrast was enhanced, and the average fluorescence intensity was measured using built-in ImageJ functions [16].

## 3. Results and Discussion

### 3.1. Primary Human Hepatocyte-Only Culture and Co-Culture of Hepatocytes and BAECs

**Figure 2:**
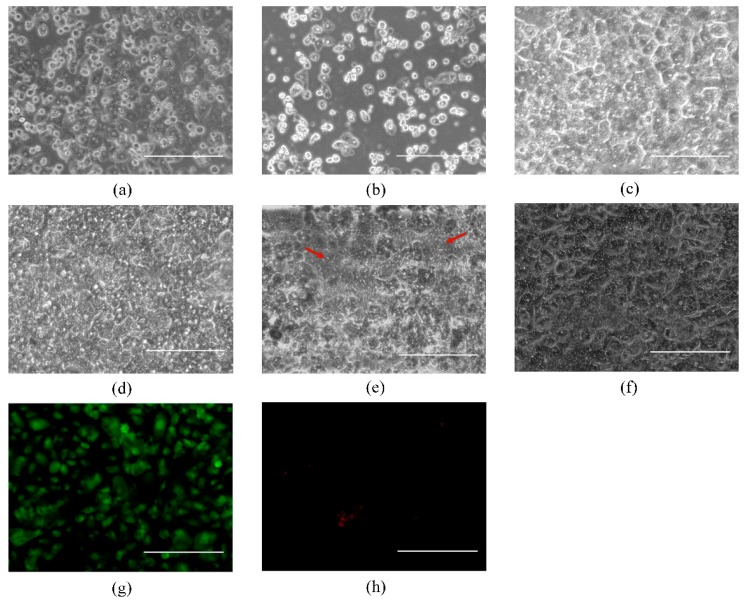
Primary human hepatocyte morphology when cultured in microchannels. Primary human hepatocytes-only, cultured in a single microchannel under static culture conditions at day 1 (**a**); and day 5 (**b**); Primary human hepatocytes when hepatocytes-only were cultured on the membrane in a dual microchannel under flow conditions at day 1 (**c**); day 5 (**d**); and day 13 (**e**). Arrows indicate empty space. Primary human hepatocytes when hepatocytes were co-cultured with BAECs in a dual microchannel under flow conditions at day 26. Bright field image of cells that were subsequently analyzed with the LIVE/DEAD Viability/Cytotoxicity Kit (**f**); green fluorescent image of LIVE/DEAD analysis, indicative of live cells (**g**); and red fluorescent image of LIVE/DEAD analysis, indicative of dead cells (**h**) of hepatocytes (stained with LIVE/DEAD Viability/Cytotoxicity Kit). Scale bar: 200 μm.

Primary human hepatocytes were cultured in the absence of other cells in a single microchannel under static culture conditions; these hepatocytes maintained their polygonal morphology for about 3 days, after which this morphology was lost (Figure 2a,b). When primary human hepatocytes-only were cultured on a microporous membrane of a dual-PDMS microchannel under continuous perfusion, these hepatocytes maintained a normal phenotype in the microchannel for about 5–7 days after seeding, which was longer than hepatocytes that were cultured alone under static conditions (Figure 2c,d). However, these hepatocytes also lost their characteristic polygonal morphology starting at about 7 days in culture; the hepatocytes began to detach from the microporous membrane, resulting in large empty spaces (Figure 2e). Overall, these observations indicated that continuous flow culture conditions provide primary hepatocytes with a better culture environment than static culture conditions, enabling primary hepatocytes to maintain their morphology for a longer but still limited period of time.

In order to determine whether the co-culture of primary human hepatocytes and endothelial cells in the microchannel facilitates longer term survival and maintenance of the differentiation status and function of culture primary human hepatocytes, we made use of BAECs in this proof-of-concept analysis; BAECs replaced LSECs in our model. Although BAECs are bovine in origin, we chose this stepwise strategy because human LSECs are considerably more difficult to obtain and cannot be passaged. Moreover, the use of BAECs did not preclude our subsequent HBV replication studies, which only rely on the presence of differentiated, normal, human hepatocyte, although it is clear that for future optimization of this model, primary human LSECs in a co-culture of primary human hepatocyte would be the most physiologically relevant combination.

When primary human hepatocytes and BAECs were co-cultured in our microchannel platform by layering on opposite sides of the microporous membrane, the cells formed a confluent monolayer and the primary human hepatocytes maintained their normal hepatocyte morphology for at least three weeks (Figure 2f). Primary human hepatocytes at the bottom and BAECs at the top were layered in the microchannel. To further investigate the extended viability of primary human hepatocytes co-cultured with BAECs, we continued to observe the co-cultured cells for 26 days and also assessed the viability of the hepatocytes using the LIVE/DEAD Viability/Cytotoxicity Kit (Molecular probes). Figure 2g,h show the green fluorescent (live cells) and red fluorescent (dead cells) images of primary human hepatocytes. In contrast with primary human hepatocytes-only culture, this result indicates that primary human hepatocytes remain viable for at least 26 days when co-cultured with BAECs in microchannels under flow conditions. In addition, this result agrees with the findings from others that primary hepatocytes show better viability and function when co-cultured with other cell types, such as fibroblasts [13,37,38]. However, we did note that the cells did not maintain a confluent monolayer over the entire surface of the microchannel at day 26, and partial detachment of the cells was noticed.

### 3.2. Infection of Primary Human Hepatocytes with Recombinant Adenoviruses and Expression of HBV Core Antigen

**Figure 3:**
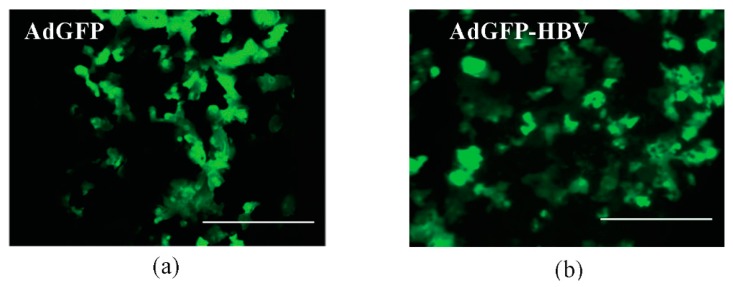
Primary human hepatocytes infected with recombinant adenovirus. (**a**) Green fluorescence image of primary human hepatocytes infected with AdGFP in the single microchannel (day 4 after cell plating); (**b**) Green fluorescence image of primary human hepatocytes infected with AdGFP-HBV in a dual microchannel (day 5 after cell plating). Scale bar: 400 μm.

In our human liver model, only about ten-thousand cells were plated on the small dimensions of the microchannel surface. We next investigated if the small number of primary human hepatocytes cultured in this limited area could be infected with viruses, providing supportive evidence for the use of our model for studying viral infections of hepatocytes, such as an HBV infection, and potential mechanisms that link these viral infections to liver disease. For this investigation, we first used infection with recombinant adenovirus instead of direct infection of primary human hepatocytes with HBV. This is because infection with AdGFP can provide preliminary information regarding viral infection of primary hepatocytes by checking the expression of GFP in the infected cells, such as how many virus infected-hepatocytes are in the microchannel. Initially we tested this in the primary human hepatocytes-only culture in the single microchannel. We infected hepatocytes in the microchannels with AdGFP on day 2 after plating. The virus was incubated in the microchannel for approximately 16 h, and then the medium in the microchannel was removed, cells were washed, and fresh hepatocyte medium was added. Primary human hepatocytes infected with AdGFP expressed GFP for at least 48 h following infection (Figure 3a, green fluorescence image) [39]. The infection efficiency of AdGFP resulted in approximately 69.4% of primary human hepatocytes expressing GFP; cells were imaged by fluorescence microscopy and analyzed by ImageJ for quantification of fluorescence intensity. As a result, we verified that the small number of primary human hepatocytes in the limited microchannel area were infected with the virus with about 70% of viral infection efficiency. Result shown in Figure 3a is from a single microchannel; similar infection efficiency was noted in a replicate sample.

**Figure 4:**
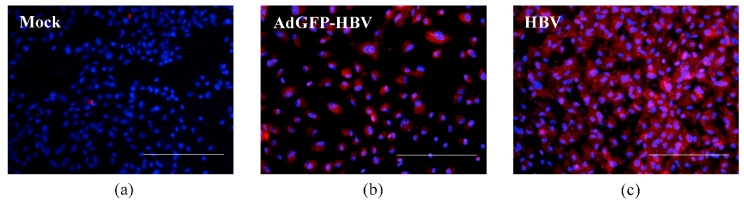
Immunofluorescence staining of HBcAg in primary human hepatocytes infected with HBV. The DAPI nuclear staining dye for the same set of cells was used for DNA staining. (**a**) Control (Mock): primary human hepatocytes co-cultured with bovine aortic endothelial cells (BAECs) without viral infection under flow conditions in the dual microchannel on day 14; (**b**) AdGFP-HBV was used as a control to ensure that the hepatocytes were viable, susceptible to a viral infection, and capable of expressing HBV proteins. HBcAg in AdGFP-HBV infected hepatocytes was detected with Hepatitis B core antibody (HBcAb) at day 14; (**c**) Direct infection with HBV. HBcAg in HBV-infected hepatocytes was detected with HBcAb at day 14. Scale bar: 200 μm.

We next sought to determine whether HBV proteins could be expressed from the HBV genome in hepatocytes in our microchannel system. We specifically tested expression of the HBV core antigen as a surrogate marker of HBV protein expression. We infected hepatocytes in the microchannel with a recombinant adenovirus that encoded GFP as well as a cDNA of the HBV genome (AdGFP-HBV). Expression of all HBV proteins from the cDNA is controlled by HBV-specific transcription promoters and relies on the differentiation status of the hepatocytes. Our goal was to show expression of HBV proteins controlled by an endogenous HBV transcription promoter in our co-culture system and we initially used the AdGFP-HBV because of the high efficiency of adenovirus infection of hepatocytes. Since this requires a longer time period for HBV proteins to be detectable hepatocytes, as compared to GFP expression, for these studies with AdGFP-HBV, we used primary human hepatocytes co-cultured with BAECs in the dual microchannel system. Hepatocytes were first added to the microchannels, followed by BAECs on the opposite of the membrane, and the hepatocytes were infected with AdGFP-HBV on day 2 after hepatocytes plating [8,40]. The microchannels were then connected to a continuous perfusion system. The cells infected with AdGFP-HBV on day 2 after plating maintained a GFP expression for 3 days after infection (Figure 3b). Analysis and quantification of the GFP expression using ImageJ demonstrated an infection efficiency of approximately 56.5% of the primary human hepatocytes by AdGFP-HBV. This efficiency is a little lower compared to the infection efficiency of AdGFP. However, this infection efficiency is consistent with results of our previous studies, in which we showed that adenovirus infection efficiency of primary rat hepatocytes ranges from 30% to 70% [20]. Approximately 12 days after infection with AdGFP-HBV, the microchannels were disassembled and Hepatitis B core antigen (HBcAg) was detected as described in the Material and Methods section (compare Figure 4a,b). Approximately 73.3% of the hepatocytes expressed HBcAg, as indicated by immunofluorescence analysis; the greater number of hepatocytes expressing HBcAg, as compared to GFP expression, may indicate that secreted HBV could infect neighboring hepatocytes that were initially uninfected by AdGFP-HBV and that these hepatocytes now express HBcAg. Overall, these initial studies indicated that the small amount of primary human hepatocytes cultured in the microchannel could be infected with the recombinant adenovirus and express HBV proteins, where expression of these proteins was controlled by HBV transcription promoters that are sensitive to the differentiation status of hepatocytes.

### 3.3. HBV Infection and Replication in Primary Human Hepatocytes in Microchannels

We next sought to demonstrate the utility of our system for studies with hepatotropic viruses by testing whether primary human hepatocytes co-cultured with BAECs in our microchannel system could be directly infected by HBV. HBV infection of primary human hepatocytes is sensitive to the differentiation status of the hepatocytes and the expression of the HBV cell surface receptor, which is known to be lost when human hepatocytes de-differentiate [41]. In addition, HBV typically replicates at low levels in hepatocytes requiring prolonged incubation of differentiated hepatocytes, so as to achieve levels of replication that are sufficient for detection as secreted HBV virions [5,42]. We directly infected primary human hepatocytes with HBV on day 2 after plating and then analyzed HBV replication and expression of HBV core protein in this system; expression of HBcAg was also used as one indicator of infection efficiency. The HBcAg in HBV-infected primary human hepatocytes was detected with Hepatitis B core antibody (HBcAb) at day 14 (compare Figure 4a,c). HBcAg was not detected in the control primary human hepatocytes (Figure 4a). This analysis demonstrated that approximately 72.9% of hepatocytes in the microchannel were HBV-infected; the analysis of immunofluorescent images was done using ImageJ. Figure 4a,c are representative of one experiment, but similar results were obtained in a replicate analysis.

**Figure 5:**
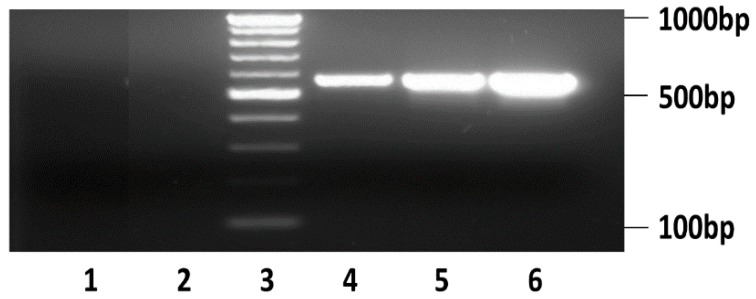
PCR analysis of supernatant samples from the co-culture of primary human hepatocytes and BAECs on the liver-sinusoid-on-a-chip was performed for the detection of secreted HBV from the infected hepatocytes. Lane 1: water; Lane 2: mock (supernatants collected from hepatocytes without viral infection at day 22); Lane 3: DNA ladder; Lane 4: supernatants from hepatocytes infected with HBV (day 22); Lane 5: supernatants obtained from HepG2.215; and Lane 6: positive control of HBV plasmid. As a positive control for a HBV gene expression analysis, a plasmid containing a cDNA of the HBV genome was used.

To further determine whether HBV can both infect and replicate in the cultured primary human hepatocytes co-cultured with BAECs in our microchannel system, we analyzed secretion of replicated HBV into the cell culture medium by collecting cell culture supernatants at the indicated time points from the microchannels. HBV genomes were isolated from the supernatants collected from the cells, and the genome was detected by PCR and gel electrophoresis as described in Section 2.6. HBV genomes from the HBV-infected hepatocytes were detected (Figure 5, Lane 4) and were identical in size to the band from the positive control (Figure 5, Lane 6). HBV was not detected in the negative control (Figure 5, Lane 1). Also, HBV was not detected in supernatants collected from uninfected control primary human hepatocytes (Figure 5, Lane 2). As an additional positive control for the isolation of HBV from cell culture supernatants, we collected supernatant from HepG2.215, which is the established cell line that produced infectious HBV. A HBV-specific PCR product was detected in the supernatant from HepG2.215 cells (Figure 5, Lane 5). These HBV-specific PCR products were identical in size to the band from the positive control (Figure 5, Lane 6). Thus, the results of this analysis confirmed that HBV-specific PCR products were detected only in cell-culture supernatants from positive controls or from primary human hepatocytes infected with HBV. Importantly, HBV is hepatotropic, and the expression of HBV RNA transcripts and HBV replication is dependent on the expression of hepatocyte-specific factors. Hence, the detection of HBV replication in our microfluidic platform serves as a confirmation that the differentiation status and functions of hepatocytes were retained throughout the course of our study.

Although our experiments have a drawback in terms of the species discrepancy caused by co-culturing human hepatocytes and BAECs, these studies demonstrate the utility of systems in which the long-term maintenance of human hepatocyte differentiation status can be retained so that the system can be used for studies of human hepatotropic viruses. In future studies, we will need to validate the HBV replication study described here in a co-culture system of primary human hepatocytes and primary human liver sinusoidal endothelial cells. Moreover, from the aspect of the pathophysiology of viral infection in vivo, it may also be necessary to infect hepatocytes with HBV through the endothelial cell layer by introducing HBV into the microchannel where endothelial cells are cultured. However, even considering the requirement of these future modifications, we have presented a human-liver-sinusoid-on-a-chip model, wherein primary human hepatocytes that are co-cultured with BAECs in the microfluidic platform can be used in HBV replication studies. Moreover, we believe that our microfluidic co-culture system can be extended to long-term studies involving other viral infections of the human hepatocytes.

## 4. Conclusions

We have developed a human liver model where primary human hepatocytes and BAECs are co-cultured on opposite sides of a microporous membrane in a dual microfluidic platform. Primary human hepatocytes co-cultured with BAECs retained their morphology and viability for up to 26 days. The primary human hepatocytes cultured in the microfluidic channel were infected with HBV. The HBcAg of the primary human hepatocytes infected with HBV was detected with an anti-HBcAg antibody by immunofluorescence. Moreover, secreted HBV was detected by measuring cell-secreted HBV DNA from the supernatants collected from the HBV-infected hepatocytes. The ability to detect HBV replication in our microfluidic platform serves as a confirmation that the differentiation and functional status of hepatocytes was retained throughout the course of our experimentation.

Overall, we have presented a novel human-liver-sinusoid-on-a-chip model system wherein primary human hepatocytes are co-cultured with BAECs in the microfluidic channels and have demonstrated the utility of this model system for HBV-related studies. We believe that our microfluidic culture system can be extended to long-term studies involving viral infections of primary human hepatocytes that were previously not possible due to the rapid loss of function of primary hepatocytes in conventional cell-culture systems. For example, it may be possible to apply our liver model to study chronic HBV infection that is characterized by the persistence of HBsAg for the long-term as well as the persistence of HBV cccDNA. Finally, we believe that our human-liver-sinusoid-on-a-chip could also find numerous applications in other liver-related research and drug development.
